# Increasing Residential Radon Testing Through Outreach to Families of Newborn Children in Pennsylvania: Evaluation of Intervention Outcomes, 2002–2023

**DOI:** 10.1016/j.focus.2025.100372

**Published:** 2025-05-23

**Authors:** Tarya Pillay, Mia N. Ray, Rebecca Bascom, Jennifer L. Moss

**Affiliations:** 1 Department of Family and Community Medicine, Penn State College of Medicine, The Pennsylvania State University, Hershey, Pennsylvania; 2 Department of Medicine, Penn State College of Medicine, The Pennsylvania State University, Hershey, Pennsylvania; 3 Department of Public Health Sciences, Penn State College of Medicine, The Pennsylvania State University, Hershey, Pennsylvania

**Keywords:** Radon, lung cancer, newborn, parents, public health, RE-AIM

## Abstract

**Introduction:**

Few people undertake residential testing for radon, despite its known impact on lung cancer risk. Motivated by new parents’ interest in residential hazards, the Pennsylvania Department of Environmental Protection’s Newborn Radon Testing Project distributes free radon testing vouchers to new parents. Impacts on radon testing are unknown.

**Methods:**

The authors evaluated the Newborn Radon Testing Project (2002–2023) utilizing the Reach, Effectiveness, Adoption, Implementation, and Maintenance framework. Data came from project records as well as Pennsylvania Department of Health Birth Statistics. The authors analyzed outcomes according to Reach, Effectiveness, Adoption, Implementation, and Maintenance.

**Results:**

Among 3,093,704 babies delivered in Pennsylvania hospitals from 2002 to 2023, 24,165 (0.78%) parents requested radon testing kits through the project (Reach). Of these, 11,556 (47.7%) completed the kits, and 41.8% had radon above 4 picocuries per liter, including 18.2% with very elevated radon (>10.0 picocuries per liter) (Effectiveness). Among eligible hospitals, 87 of 107 (81%) partnered with the project (Adoption), and average program costs were $14,507 per year or $29 per analyzed kit (Implementation).

**Conclusions:**

The Newborn Radon Testing Project holds promise for identifying homes with elevated indoor radon levels. Efforts are needed to increase participation and monitor remediation among new parents.

## Highlights

• Parents of newborns may be especially motivated to reduce residential radon levels.
• From 2002 to 2023, 24,165 parents of newborns requested free radon testing kits.
• Half of parents completed testing, of whom 42% had elevated residential radon.
• Radon testing and remediation among parents could benefit population health.

## INTRODUCTION

Radon is a colorless, odorless, naturally occurring radioactive gas found in uranium-rich rocks and soil that enters into buildings through cracks and openings in the foundation.^1^^,^^2^ As it decays, radon breaks down into carcinogenic byproducts, which are aerosolized and inhaled, causing damage to lung tissue and DNA.^3^ Radon exposure contributes to 13% of lung cancer deaths in the U.S. annually, and it is the leading cause of lung cancer among nonsmokers.^2^^,^^4^^,^^5^ Children may be especially susceptible to the effects of radon inhalation because of their faster breathing rates and more permeable airway epithelium,^6^ which preliminary evidence suggests can increase the risk of inflammation, asthma, and leukemia.^7^, ^8^, ^9^, ^10^

To reduce the risk of radon-associated lung cancer, the Environmental Protection Agency (EPA) recommends residential radon testing and remediation.^11^ Radon testing involves placing a measuring device in the home (typically for 2–4 days) and then sending it to a laboratory for analysis.^12^ The EPA recommends remediation if the test indicates that average radon levels exceed 4 picocuries per liter (pCi/L). Remediation generally involves sealing cracks in the building’s foundation and installing a vent pipe and a fan.^5^^,^^13^

Despite radon being a known carcinogen and public health threat, testing and remediation are low.^14^ Barriers to testing and remediation include lack of knowledge about radon, low perceived risk from radon exposure because it is a naturally occurring substance, high anticipated costs of remediation, and insufficient legal regulation.^14^, ^15^, ^16^ Previous research studies aimed at increasing radon testing have focused on information dissemination through realtors, primary care providers, and a smartphone app, all of which resulted in increased testing.^17^, ^18^, ^19^, ^20^ However, these interventions have generally reached a small and select group of participants, without widespread, population-based outreach.

This study seeks to evaluate the Newborn Radon Testing Project, a program developed by the Pennsylvania Department of Environmental Protection to increase residential radon testing. Program activities focused on outreach and information dissemination to families of newborn children, and the authors present outcomes of the program from 2002 to 2023. This analysis is guided by the Reach, Effectiveness, Adoption, Implementation, and Maintenance (RE-AIM) evaluation framework. The findings from this evaluation could have implications for other state government- or healthcare-based programs to increase radon testing.

## METHODS

### Setting

Pennsylvanians are particularly vulnerable to radon exposure because of the statewide prevalence of uranium-rich rocks and soil as well as other geologic and climactic factors that increase radon exposure. Among 67 counties in Pennsylvania, 48 (72%) are classified by EPA as Radon Zone 1 (i.e., highest levels of potential radon exposure), and 37.7% of all households in the state have radon levels exceeding the EPA’s 4 pCi/L action level when measured at basement level.^2^^,^^21^

### Program Description

The Newborn Radon Testing Project has been managed by the Pennsylvania Department of Environmental Protection, Bureau of Radiation Protection (BRP), Radon Division since 1996. The project focuses on radon information dissemination to parents and guardians (referred to as parents in the remaining parts of this paper) when they are discharged from certain hospitals in Pennsylvania after the birth of a newborn child. Its goals are to increase residential radon testing and remediation as an effective way to prevent lifelong disease and disability associated with environmental exposures. This program was initially motivated by new parents’ increased awareness of safety hazards and corresponding motivation to prevent health risks, which has been demonstrated previously.^22^^,^^23^

Hospitals in Pennsylvania can enroll into the program, and then the BRP sends them packets that include the “Pennsylvania Residents’ Guide to Radon”^24^ and vouchers for free radon testing. This 24-page guide includes information on the health risks associated with residential radon exposure, how radon accumulates in the home, and instructions for home testing and remediation. After enrolled hospitals receive the radon packets, hospital staff provide these packets to parents of newborns when they are discharged from the hospital after childbirth. Parents who are interested in radon testing mail the completed voucher for free radon testing to BRP, which provides parents’ contact information to their partner radon testing laboratory. The laboratory sends, by mail, a radon testing kit and instructions for use to interested parents. After using the test kit, parents return it to the laboratory for analysis, and the laboratory subsequently communicates results to the parents and to BRP. In addition to the radon test results, the report from the laboratory also includes recommendations for remediation, including resources to offset costs.

Each year, BRP generates deidentified, aggregated summary reports of the Newborn Radon Testing Project activities, including hospital enrollment, participation from parents, and results and costs of radon testing.

### Evaluation Approach and Analysis

This evaluation of the Newborn Radon Program is guided by RE-AIM.^25^ RE-AIM is a commonly used evaluation framework outlining 5 domains of key intervention outcomes.^26^ Data for this evaluation were gathered from the annual Newborn Radon Testing Project reports as well as administrative data on the annual number of newborn children born at hospitals from the Pennsylvania Department of Health Birth Statistics.^27^

Reach is the number and representativeness of program participants.^28^ To measure reach, the authors summarized the number of newborn babies delivered at hospitals in Pennsylvania from 2002 to 2023; these individuals could be considered the universe of potential program participants. Then, the authors summarized the number and proportion of radon testing kits distributed to quantify program participation. Owing to data availability, the authors were not able to assess representativeness.

Effectiveness is the impact of an intervention on outcomes of interest.^28^ To measure effectiveness, the authors assessed the number and proportion of radon testing kits returned/analyzed (among those distributed). In addition, the authors assessed the results of the testing kits analyzed through the Newborn Radon Testing Project. Specifically, the authors summarized the number and proportion of tests that indicated acceptable levels of radon exposure (i.e., <4.0 pCi/L), elevated exposure (i.e., 4.0–9.9 pCi/L), and very elevated exposure (i.e., >10.0 pCi/L) or void results across the study period. In addition, because radon concentrations may vary across the calendar year, the authors examined whether season was associated with having elevated radon exposure (≥4 pCi/L) using chi-square tests.

Adoption is the number and representativeness of program settings.^28^ To measure adoption, the authors assessed the number of hospitals in Pennsylvania that were eligible to participate in the Newborn Radon Testing Project (for evaluation purposes, eligibility was defined as hospitals with >12 births recorded in a given year), overall and yearly; these hospitals could be considered the universe of potential program settings. Then, the authors summarized the number and proportion of participating hospitals as well as the number of radon information packets requested by hospitals each year. Owing to lags in data reporting, data on the number of potentially eligible hospitals was only available for 2002–2021.

Implementation reflects the quality of program delivery (e.g., fidelity, consistency, time, and cost).^28^ Owing to data availability, the measurement of implementation focused only on costs of testing; other indicators of implementation and of costs (e.g., personnel time) could not be assessed. The authors assessed total costs of radon testing as part of the Newborn Radon Testing Project, overall and yearly. In addition, the authors evaluated the cost of radon testing per testing kit returned by parents.

Maintenance is the institutionalization of the program.^28^ Because the Newborn Radon Testing Project has been supported by the Pennsylvania Department of Environmental Protection for more than 20 years, this domain is not relevant for the current evaluation.

Data management and analyses were conducted using Excel and SAS, Version 9.4 (SAS Institute, Cary, NC). This evaluation was exempt from ethics review because it involved analysis of aggregated, deidentified, and publicly available data.

## RESULTS

### Reach

Between 2002 and 2023, 3,093,704 newborn babies were delivered at hospitals in Pennsylvania (Table 1). Among this pool of potential study participants, 24,165 parents (0.8%) received the radon information packets and requested a testing kit (annual range: 212–2,528).

**Table 1:** Measures of Reach, effectiveness, Adoption, and Implementation for Pennsylvania’s Newborn Radon Testing Project

Measure	Total	Yearly average	Range across years
Reach			
Babies delivered at hospitals in Pennsylvania	3,093,704	154,685	(130,730–150,322)
Radon test kits distributed to parents	24,165	1,098	(212–2528)
Radon test kits returned by parents	11,556	525	(105–1272)
Effectiveness			
% of kits returned among kits distributed	47.7%	46.7%	(30.0%–78.8%)
Adoption			
Eligible hospitals in Pennsylvania^a^		107	(84–139)
Participating hospitals		87	(51–116)
Radon information packets requested by hospitals	462,884	20,125	(6,750–44,420)
Implementation			
Costs of radon testing	$333,670	$14,507	($2,170–$45,627)
Costs of radon testing/test kit returned by parents	$29		
Costs of radon testing/result above the EPA action level	$68		

a Eligible hospital estimates span from 2002 to 2021. EPA, U.S. Environmental Protection Agency.

### Effectiveness

**Figure 1:**
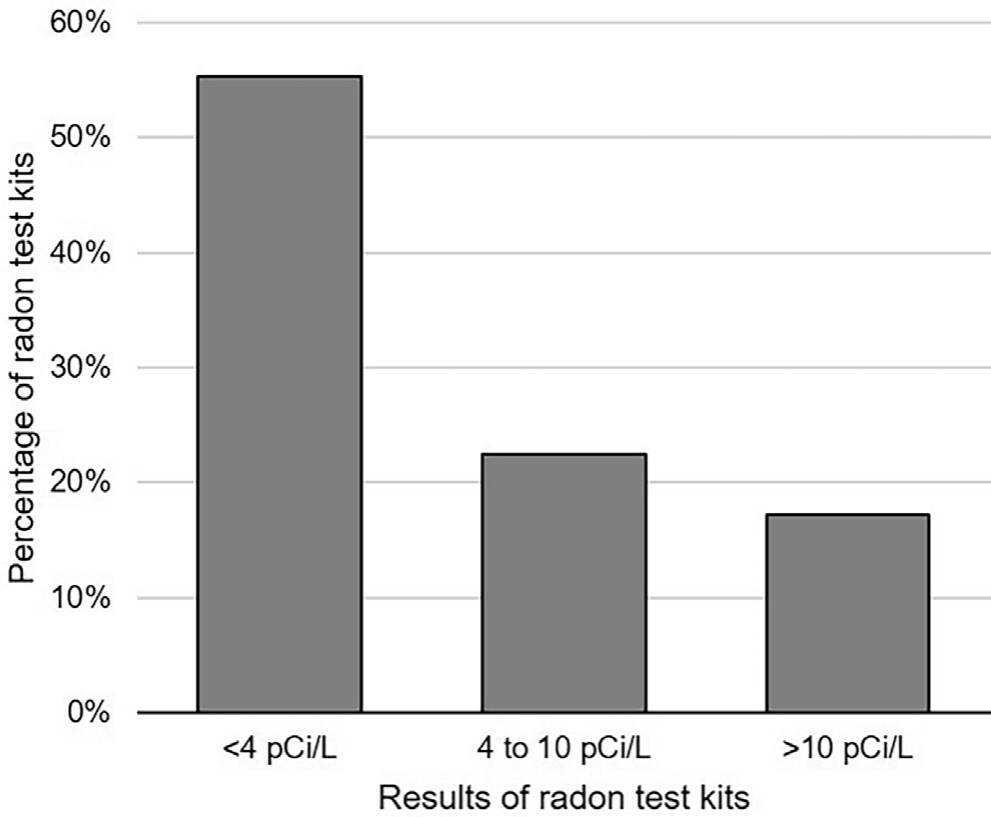
Outcomes of radon testing kits distributed through Pennsylvania’s Newborn Radon Testing Project.

Among the parents who requested it, 11,556 (47.7%) used and returned the radon testing kit to the laboratory (annual range: 30.0%–78.8%). Among these kits, 589 had void results. Among the 10,967 remaining kits with valid results, 58.2% had acceptable radon levels (<4.0 pCi/L). However, 23.6% had elevated levels (4.0–9.9 pCi/L), and 18.2% had very elevated levels (>10.0 pCi/L) (Figure 1). That is, 41.8% indicated residential radon levels above the EPA action level. The percentage of tests with a result indicating elevated radon levels varied across seasons (from 37% in summer months [June, July, August] to 46% in spring months [March, April, May], chi-square=39.07, *p*<0.001).

### Adoption

Each year (2002–2021), an average of 107 hospitals were potentially eligible (i.e., had >12 births in a given year) to participate in the Newborn Radon Testing Project, and an average of 87 (81%) of these hospitals actually participated (Table 1). Across the entire study period (2002–2023), participating hospitals requested a total of 462,884 radon information packets or 20,125 packets per year.

### Implementation

For the entire study period (2002–2023), incurred costs for radon testing through the Newborn Radon Program totaled $333,670 or an average of $14,507 per year (Table 1). The average cost per test for laboratory-based analysis was $29.

## DISCUSSION

From 2002–2023, the Newborn Radon Testing Project in Pennsylvania had low participation from eligible parents of newborns, with <1% of hospital births with a corresponding request for a radon testing kit. However, of the parents who engaged with the program, nearly half returned the provided test kits. More than 40% of the results from returned kits indicated residential radon levels equal to and greater than the EPA’s action level of 4 pCi/L, including 18% of results indicating very high levels (>10 pCi/L). The program holds promise for identifying elevated radon levels; however, additional efforts are necessary to increase the reach of the program to a larger proportion of eligible parents and to ensure that disadvantaged communities are included in the program’s reach. Further research should examine parents’ mitigation behaviors after an elevated test result as well as the impact of the program on long-term health outcomes. The Newborn Radon Testing Project also provides a model that other states could adopt to raise awareness and increase residential radon testing.

Of the Pennsylvania hospitals that routinely offer labor and delivery services, most were enrolled in the Newborn Radon Testing Project. Importantly, the project was not advertised to all hospitals in the state because, owing to resource limitations, the program initially targeted hospitals in EPA Radon Zone 1 and Zone 2 counties (i.e., excluding Philadelphia county, which is the state’s most populous county and the only county classified as a Radon Zone 3).^21^ The extent to which they engaged with the program is unclear, such as whether requested newborn packets were passed out to all parents at discharge (i.e., fidelity to the protocol). Optimally, the packet would have been included in all take-home bags for new parents, but the number of hospital-requested packets was far fewer than the number of births. Future research should use implementation science theories or frameworks to examine hospital-level participation, engagement, and fidelity.

However, of the parents who received these packets and requested testing kits, almost half (47.7%) returned the completed tests, suggesting a relatively high level of engagement among parents who were aware of the project. This testing completion rate among people who receive radon kits is similar to results from other interventions taking place in regions with high radon exposure: 48% in Kentucky^20^ and 52% in North Dakota.^19^ Thus, it appears that increasing initial participation (either by providing free kits directly or increasing the percentage of people who request a free kit) may be the key determinant of success of a radon testing intervention. Among parents of newborns, parents who requested a testing kit likely vary systematically from parents who did not request a kit, and they may exhibit higher risk perception or greater residential hazard anxiety.^29^ Possible reasons for parental nonparticipation include competing demands for time and attention during the newborn period. Previous research indicates that time constraints associated with maternal responsibility, including work, household management, and infant care, served as significant barriers to behavior change in the postnatal period.^30^ Other barriers to participation may include renting versus owning a home because individuals who rent their home may not view radon testing as their responsibility, and Pennsylvania law does not require landlords to remediate residences for radon if high levels are detected. It is possible that behavioral science techniques (e.g., including a call to action) could increase the impact of radon-related information communicated to parents in such a way as to increase engagement.^31^ Yet, the number of completed radon tests is itself notable, and it is unknown what portion of the 11,556 parents who underwent radon testing through this program would have completed testing without outreach through the program.

The current analysis of reach and adoption is limited by lack of data on the number of parents who had more than 1 child during the study period and were double counted in the analysis. In addition, the authors do not have data on the number of parents receiving booklets who had previously tested their homes for radon and were therefore not interested in participating. Potential next steps for newborn radon testing in Pennsylvania include prospective research with parents to identify possible barriers to participation. The reach of the program could be expanded by leveraging other perinatal touchpoints, such as obstetricians, pediatricians, and family practitioners, in the prenatal and postnatal periods; for example, one study found that almost 15% of participants who received vouchers for free radon testing through primary care offices completed testing within the following year.^32^ Although the Newborn Radon Testing Project’s reach was relatively low, its effectiveness for detecting elevated residential radon exposure is notable. Furthermore, among the completed tests, almost half had results indicating that their residential radon exposure was above the EPA recommended action level, including 18% with very high radon exposure levels. The frequency of actionable results is equivalent to or slightly higher than the estimate that 40% of all residences in Pennsylvania have radon levels exceeding the 4 pCi/L benchmark.^2^

Unfortunately, data are unavailable about whether parents with elevated radon levels undertook remediation, and future studies using personally identifiable information should explore this process. Notably, the report communicating the results of their radon test included information about resources to help parents access radon remediation (including financial support for eligible households). Furthermore, the costs for this program average $29 per completed radon testing kit, which is a relatively low cost in comparison to potentially avoidable healthcare costs from radon-associated health problems but is generally higher than many commercially available radon testing kits. However, additional research is needed to fully understand radon remediation and cost savings for the program.

This study represented a pragmatic evaluation of a real-world, government-supported public health intervention. The evaluation faced certain data limitations, including lack of data on parent characteristics. Furthermore, owing to processes for tracking expenses at Department of Environmental Protection, a small portion of the costs for radon testing through the Newborn Radon Testing Project was actually attributed to tests distributed through other outreach events (e.g., realtor events). The authors could not disentangle these sources of radon testing kits, so, conservatively, the authors included all of incurred costs in the current program evaluation. In reality, the costs of the project were lower than the data the authors presented. These types of limitations often impact real-world programs.^33^^,^^34^ Although highly controlled research experiments might be able to overcome some of these challenges, the external validity of the program evaluation is high.

This study had several strengths. The Newborn Radon program is a government-supported program with statewide coverage, increasing the potential reach of this program and potential transportability to other state contexts. This program targets an understudied exposure (i.e., radon) that has established, deleterious impacts on health; thus, the program and its evaluation address an unmet need for public health intervention. In addition, the program evaluation adds to the research literature on parents’ health behavioral change during the perinatal period, a time of increased motivation to improve residential health and safety.

### Limitations

In terms of limitations, as noted, individual-level data on program participants were not available. This limitation precludes analysis of (1) correlates of radon testing as well as (2) whether parents living in residences with elevated radon exposure undertook remediation. The Newborn Radon Testing Project used to include a follow-up survey delivered to parents with elevated radon test results, but this survey was deemed an invasion of privacy in 2011 and could no longer be distributed; information from that survey could have addressed this limitation. Similarly, the authors did not have data on actual distribution of radon information packets from hospitals to parents, limiting the analysis of hospital and parent engagement. In addition, other constructs from the RE-AIM framework were excluded from this analysis, for example, fidelity and consistency. Future research and evaluation should address these limitations.

## CONCLUSIONS

The Newborn Radon Testing Project is a model for the dissemination of radon information and free testing kits to a population (i.e., parents of newborns) that is highly motivated to reduce residential hazards. The program has high participation among eligible hospitals; however, additional efforts are necessary to increase the reach and adoption of the program to maximize participation and impact. For parents who participate, the Newborn Radon Testing Project is effective at detecting residential radon exposure above the EPA’s action level, which is a key step in facilitating population-level radon mitigation and reducing exposures that could lead to lung cancer.

In summary, the authors evaluated the Pennsylvania Department of Environmental Protection’s Newborn Radon Testing Project using the RE-AIM framework. Study findings demonstrate that although reach and adoption were low, the program was successful in identifying homes with elevated residential radon exposure. As such, other states could use similar methods to increase radon testing among their populations.
